# Influence of Ground Calcium Carbonate Waste on the Properties of Green Self-Consolidating Concrete Prepared by Low-Quality Bagasse Ash and Rice Husk Ash

**DOI:** 10.3390/ma14154232

**Published:** 2021-07-29

**Authors:** Pusit Lertwattanaruk, Natt Makul

**Affiliations:** 1 Faculty of Architecture and Planning, Thammasat University, Pathumthani 12121, Thailand; 2 Faculty of Industrial Technology, Phranakhon Rajabhat University, Bangkok 10220, Thailand; natt@pnru.ac.th

**Keywords:** green self-consolidating concrete (gSCC), ground calcium carbonate waste (GCW), bagasse ash (BA), rice husk ash (RHA), workability, mechanical properties

## Abstract

Bagasse ash (BA) and rice husk ash (RHA) are by-products from electricity power plants. Ground calcium carbonate waste (GCW) is the by-product of the mining of calcium carbonate (CaCO_3_) in the color pigment manufacturing industry. Both BA and RHA are classified as low-quality pozzolanic materials, differing from GCW, which contains a high calcium oxide (CaO) content that leads to products equivalent to the hydration reaction. Therefore, GCW is likely able to improve the properties of self-consolidating concrete (SCC) incorporating BA and RHA. This paper discusses the production of green self-consolidating concrete (gSCC) and identifies the benefit of using GCW in gSCC prepared by triple combined GCW (10 and 20 wt%), BA (10, 20, and 30 wt%), and RHA (20 wt%). The results indicate that the majority of the gSCC retain acceptable flowability. The differences in the levels of gSCC substitution and the V-funnel flow results show general correlations with the increase in GCW. The gSCC prepared by 10 wt% GCW associated with 10 wt% BA and 20 wt% RHA was improved significantly. The filling and passing abilities of the gSCC were improved by using GCW. In addition, gSCC achieved mechanical property development and was able to minimize the consumption of OPC by up to 40%.

## 1. Introduction

The cement industry is becoming a severe threat to ecology due to its emission of carbon dioxide (CO_2_). The modern world is looking for alternatives so that the environment can be saved for the next generation [1,2,3,4]. To combat climate change, cement producers have tried to minimize the consumption of Portland cement through the application of supplementary cementitious materials, such as natural pozzolanic materials, to minimize emission of carbon dioxide (CO_2_) and partly substitute Portland cement in concretes.

It is well known that pozzolanic materials play an important role when incorporated in concrete production using a reduced Portland cement content. One of the conventional pozzolanic materials is fly ash obtained from thermal power plants where coal, such as anthracite, bituminous and lignite, is used as fuel to generate electricity. This by-product can potentially provide additional positive effects for concrete such as increased workability [5,6], especially for utilization in self-consolidating concrete (SCC), and also for the development of long-term compressive strength and other durability properties. Additionally, a by-product such as silica fume, originally obtained from the electric arc furnace of silicon metal or ferrosilicon alloys, can provide super-strength concrete at an early stage of hydration and pozzolanic reactions, as well as a filling effect from natural nanoscale particles. However, these materials are widespread in countries that have heavy industry such as the USA, China, and Germany. Import of these materials into other developing countries for use as raw materials in concrete production results in a higher cost of transportation. This critical issue causes other countries to pursue selective materials in local regions with lower costs of preparation for use as concrete-making materials.

One potential solution to low-cost replacement materials for Portland cement is to use the by-products from thermal power plants. In Southeast Asian countries such as Thailand, Malaysia, Vietnam, and Indonesia, biomass such as rice husk ash (RHA) [7,8], bagasse ash (BA) [9,10,11], and palm bunch are commonly used as fuel to generate electricity. The biomass ashes obtained as by-products are continuously increasing in quantity, causing waste disposal problems, air pollution, and contamination of soil and water resources [12]. Nonetheless, biomass ashes are natural materials and have intrinsic variations, causing the by-products to be correspondingly varied in both chemical composition and physical properties, resulting in varying properties of the concrete. Biomass ash mainly consists of silicon dioxide (SiO_2_), which is needed to react with calcium hydroxide (Ca(OH)_2_) from the hydration reaction of Portland cement, but the large particles of biomass hinder the reactivity of the pozzolanic reaction. Thus, the cost of grinding for reducing the particle size of biomass ash becomes an essential factor and a limitation of the use of biomass ash as cementitious materials. Typically, thermal power plants only use biomass to produce heat to achieve a certain energy level needed, rather than having a production process that facilitates other ash utilization. This has a great effect on the combustion temperature of biomass in an incineration plant, resulting in the properties of biomass ash in the hydration reaction with Portland cement. In Thailand, BA is the main biomass ash used as a raw material in the production of SCC due to the fact that SCC is a type of high-performance concrete that conventionally consumes high-volume binder materials.

Bagasse ash (BA) is a by-product obtained from the combustion of bagasse for thermal power generation in the sugar manufacturing industry; it has of late been recognized as a low-quality pozzolanic material [2]. Therefore, most BA is still disposed of in landfills daily, resulting in ecological challenges in the world. BA received from the sugar industry needs a higher water content in the mixtures of concrete production because it has high porosity and large particle sizes, leading to a low compressive strength of the concrete [11]. The compressive strength of the concrete containing ground BA improves greatly when the BA is ground into fine particles. According to Clark et al. [3], fine or ground BA can be utilized as a substitute for up to 30% of the weight of ordinary Portland cement (OPC), and the 28-day and 90-day compressive strengths of concretes can be lower than those of concretes without BA. Thus, other biomass ashes such as rice husk ash (RHA) are also possible alternative cement replacement materials.

Rice husk ash (RHA) is a possible material for use in cement substitution to achieve these improvements. Rice husk ashes obtained from burning rice hull/husk to generate power can be utilized as a cementitious material partially replacing Portland cement in the production of SCC. RHA commonly consists of up to 25% of SCC composition, which meets the compressive strength for building code specifications and workability criteria of the European Federation of National Associations Representing for Concrete [13]. Self-consolidating concretes normally require compressive strengths in the range of 28 to 35 MPa. Therefore, fine aggregate replacements have not reduced the costs of concrete production. Nevertheless, the incorporation of BA and RHA still has a limitation as Portland cement replacement materials. Furthermore, the utilization of the other fine filler materials can potentially improve the replacement rate of BA and RHA and also enhance the early-age and long-term properties of SCC. A better alternative is to use waste from another industry such as ground calcium carbonate waste (GCW) as a filler material to increase the efficiency of SCC concrete. 

More than 10,000 tons of GCW, by-products of mining of calcium carbonate (CaCO_3_) in the pigment manufacturing industry, are produced annually, and these amounts tend to increase each year [14]. The majority of these residues are disposed as wastes into the atmosphere, resulting in environmental challenges to landfills since there is only a small likelihood of their application in other uses. Subsequently, trials have attempted to utilize calcium carbonate in other applications, particularly in concrete uses [15]. It was revealed that mixtures of pozzalan material with a high calcium oxide (CaO) content and GCW that is rich in calcium hydroxide (Ca(OH)_2_) generate pozzolanic reactions, leading to end products equivalent to those produced from hydration processes of cement. According to Turuallo and Mallisa [12], mortars prepared from mixtures of fly ashes and GCW had compressive strengths of 20.9 MPa at 90 days. By weight, the optimal ratio of fly ash to GCW was 70:30. Kerkhoff [16] investigated mortars having RHA and GCW. It was reported that the optimal ratio of RHA and GCW to accomplish the greatest potential compressive strengths was 50:50 wt%. At one month, the mortar’s compressive strength was 15.6 MPa and improved to 19.1 MPa at six months [9].

This research investigates the effect of GCW in green self-consolidating concrete (gSCC) prepared by triple combined bagasse ash (BA), rice husk ash (RHA), and GCW. The low-quality BA-and-RHA gSCC was improved by using GCW that has a greater availability than other supplementary cementitious materials. The gSCC mixtures of BA and RHA might be utilized to substitute OPC in concrete works. This can help in reducing the emission of CO_2_ due to the reduction in the production of Portland cement. This can also improve the values of waste substances instead of the alternative of transferring them to landfills where they cause air and soil pollutions.

## 2. Materials and Methods

### 2.1. Materials 

#### 2.1.1. Ordinary Portland Cement (OPC)

In this research, Type I Ordinary Portland cement (OPC) was introduced as the main cementing material complying with ASTM C150 [17]. This was carried out with a percentage replacement of up to 20 wt% of GCW to accelerate the reactions between ground BA and RHA. Table 1 and Table 2 show, respectively, the physical properties and chemical components of OPC, BA, RHA and GCW.

#### 2.1.2. Bagasse Ash (BA)

In this research, the BA utilized was obtained from the sugar industry, in which it was burned at temperatures of 800–850 °C and under an oxygen content of 34.5% in the burning chamber to produce electricity. The original BA was unsuitable for applications as low-reactivity pozzolanic materials in concretes because of their high porosity and large particle sizes. Chao and Kuo [2] and Clark et al. [3] revealed that the filler effects and pozzolanic activities of industrial ashes depend on their fineness and particle size. Therefore, using a Los Angeles grinding machine (ELE International, Bedfordshire United Kingdom) for 3.0 h at a rotating speed of 33.0 rpm, the original BA was dried to reduce their moisture contents to about 1.0–2.0% (dry basis) and was ground until the particles were less than 15% by mass and retained on a 45 micrometer (µm) sieve. In addition to the BA, as-received RHA was the main by-product obtained from the electricity power plant that only used rice husk as fuel in the central part of Thailand. Its burning temperature was in the range of 650–680 °C under an oxygen content of 65.5% in the burning chamber.

**Figure 1:**
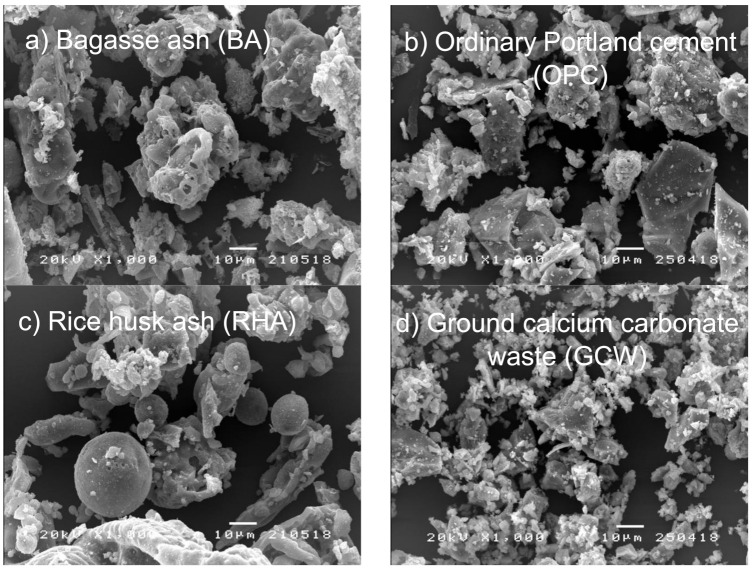
Scanning electron micrographs (1000×) of (**a**) BA, (**b**) OPC, (**c**) RHA, and (**d**) GCW.

Table 1 shows the physical properties of OPC, ground BA, RHA and GCW. The pozzolanic activity by a Chapelle test of the materials (mg CaO/g sample) was also presented. Mean particle sizes of the materials were tested 3 times each using a laser particle analyzer (Malvern Panalytical Ltd., Malvern, UK). BA has a mean particle size of 25.70 µm and is larger than the other materials. Specific gravity and percentage retained on a 45 µm sieve (No.325) were performed in accordance with ASTM C188 [18] and ASTM C430 [19], respectively. BA has a specific gravity of 2.27. The percentage retained on a 45 µm sieve of BA particles was 14.53% by mass, higher than that of the other materials. The particle image of ground BA is irregular compared to those of OPC, RHA, and GCW, as shown in Figure 1.

Table 2 shows the chemical components of ground BA, RHA and GWC obtained from X-ray fluorescence spectroscopy. While the SO_3_ and LOI amounts were 2.27% and 19.62%, respectively, the total quantities of Fe_2_O_3_, SiO_2_, and Al_2_O_3_ were 64.20%, and the main chemical composition of BA was 64.34% of SiO_2_. For a class N pozzolan, it was acknowledged that the LOI of ground BA was greater than the limit values outlined by ASTM C618 [20].

#### 2.1.3. Rice Husk Ash (RHA)

At domestic biomass power generating plants, the dust collectors collected RHA that was similar in size. The RHA was used as treated using a Los Angeles grinding machine for 3.0 h at a rotating speed of 33 rpm. The RHA had a high content of silicon oxides (SiO_2_). By mass percentages, this should be 91.42 as the X-ray fluorescences (XRFs) detected, as indicated in Table 2. Moreover, the 50% passing particle size of RHA was 17.31 µm, which makes the RHA particles larger than the OPC particles.

#### 2.1.4. Ground Calcium Carbonate Waste (GCW)

GCW is a by-product of the grinding processes of CaCO_3_. It has a high content of water [2]. As-received GCWs were dried to reduce their moisture content to about 1.0–2.0% (dry basis) for about 1 day. Using the grinding machine, the calcium carbide residues were ground until the particles were less than 10.06% by mass and retained on a 45.0 µm sieve (No. 325). Table 1 shows the physical characteristics of the GCWs. The GCWs had a mean particle size of 3.02 µm and a specific gravity of 2.42. On a 45 µm sieve (No. 325), the percentage of particles retained was 5.12% by mass. The GCW had a shape with irregular particles, as indicated in Figure 1. Table 2 reports the chemical components of the GCWs, which consist of 56.53% calcium oxide (CaO), which is the main chemical component of the GCWs. Additionally, the LOI of GCWs was 36.16%, which was high compared to other powder materials and in accordance with the study of Hoshino et al. [4], due to the fact that the LOI test was assessed by heating the sample to at least 950 °C, and CaCO_3_ decomposes to calcium oxide (CaO) and CO_2_ at temperatures above 750 °C.

#### 2.1.5. Aggregates

In this study, the coarse aggregates were crushed limestone with a water absorption of 0.40%, a fineness modulus of 7.20, a specific gravity of 2.70, and a maximum size of 19.0 mm, which indicated a suitable gradation as specified in ASTM C33 [21]. The local river sands were the fine aggregates with water absorption of 0.80%, a fineness modulus of 3.20, and a specific gravity of 2.60, complying with a gradation as specified in ASTM C33 [21].

### 2.2. Mix Proportions of Green Self-Consolidating Concrete (gSCC)

Table 3 summarizes the mix proportions for the concretes for this research. All the mixtures of gSCCs were developed with the ratios of the water–binder materials of the control SCC at 0.40 by mass and computed with constant aggregate materials (sand + ground limestone rock). The total binder content (OPC/BA/RHA/GCW) was 650 kg/m^3^. Depending on the targeted slump flow criteria, the w/b ratios of the gSCC mixtures were varied, with the specific high-range water-reducing agent (HRWR at a concentration of 45.5%) at 2.04% per 100 kg of binder materials, which was needed to obtain the target slump flows and 70.0 ± 2.5 cm [13]. OPC was substituted with BA, with cement replacements of 10, 20, and 30 wt% and RHA of 20 wt% incorporating GCW used at cement replacements of 10 and 20 wt%.

### 2.3. Sample Preparation and Testing

#### 2.3.1. Workability of gSCC

The fresh gSCCs were made by applying revolving drum-type mixers (Siam Intercorp (Thailand) Co., Ltd., Bangkok, Thailand) for fifteen minutes in this research [15]. To achieve a 70.0 ± 2.5 cm slump flow with fresh SCCs, the practical requirements are specified more clearly than those for ordinary fresh concretes. After mixing, the workability was promptly measured, which took place over a period of almost ten minutes. Based on two major abilities, the differences can be summarized:

- Filling ability: The concrete’s ability to fully fill the formworks and deform under its weight while maintaining homogeneity is known as the filling ability. Using ASTM C1611 [22], the filling ability was estimated.
- Passing ability: The concrete’s ability to flow via a confined area without overcrowding due to aggregate concretes is known as the passing ability. The concretes were tested using J-Rings to analyze the blocking behaviors and the passing ability of SCCs according to ASTM C1621 [23].

#### 2.3.2. Mechanical Properties

From all typical batches of SCCs, 21 cylinders of 30.0 cm in height and 15.0 cm in diameter were cast. The molded samples were stored in a room at 95 ± 5% relative humidity (RH) and at a temperature of 25 ± 2 °C after they were covered with a plastic sheet after casting for one day. In lime-saturated water, the SCC samples were demolded after placing and compacting them for 24 h and they were continuously cured by soaking in tap water at 25 °C until testing at 7, 28, 60, 120, 180, 270, and 360 days. The test results recorded were the averaged results obtained using three similar samples. As specified in ASTM C39 [24], ASTM C496 [25], and ASTM C469 [26], compressive strength, splitting tensile strength, and modulus of elasticity (MOE) were determined, respectively.

## 3. Results and Discussions

### 3.1. Physical Properties of Fresh gSCC

#### 3.1.1. Water Requirements Regarding w/b for Accomplishing gSCC

The gSCC mixtures with GCW incorporating different amounts of BA and RHA required a low w/b ratio as compared to without GCW, but higher than the control SCC, in order to sustain the required slump flows. It was noted that the w/b ratio for the gSCC mixtures incorporating BA varied in the range of 0.48 and 0.54; for the mixtures of gSCC with BA and RHA, it varied from 0.49 and 0.56, and the w/b ratio requirements for the gSCCs with BA and RHA incorporating GCW varied from 0.42 and 0.49. The lower SCC w/b ratio was due to the finer particle sizes of GCW and the larger specific surface areas. The required w/b ratio conversely declines when the particles of GCW dissolve in the water. This can improve the lubrication or viscosity of the SCC mixtures in order to achieve suitable flow slumps. Moreover, the distribution of smaller particle sizes improves the mixture viscosity, while the higher distribution of particle sizes improves with declining viscosity. This is because the improved OPC + GCW or OPC contents decrease the amounts of water required. Therefore, the w/b ratio is nearly constant. In summary, while maintaining the ability to flow, an increasing quantity of binder materials leads to high viscosity. For this reason, a lower w/b ratio can improve some mechanical properties. The required w/b ratio for the gSCC with BA and RHA was greater than that for SCCs without RHA, due to the high RHA particle porosity. Nevertheless, the mixtures of gSCCs with GCW achieved greater viscosity than the gSCCs mixed with BA and RHA. 

#### 3.1.2. Fresh Density

Table 4 shows the workability of the gSCC mixtures. In gSCC mixed with GCW, the fresh densities of the gSCC mixtures incorporating BA and RHA were decreased with a GCW content of 10 wt% (B0R20G10, B10R20G10, B20R20G10, and B30R20G10). The fresh densities of SCCs with GCW of 10 wt% (B30R20G10) and 20 wt% (B30R20G20) for the gSCC mixtures mixed with BA-RHA were −4.42% and −5.50%, respectively, compared to the control gSCC (B0R0G0). This is because of the lower specific gravity of GCW (2.42). For GCW (10 wt%), the fresh density of gSCCs with 10 and 20 wt% BA and 20 wt% RHA decreased by −0.96% and −2.11%, respectively. This is because BA and RHA are coarser than the OPC, but this compensated for the additional GCW filling the spaces among the OPC particles [27].

#### 3.1.3. Workability

Table 4 shows the results of the initial slump flow test for all mixtures of gSCC compared to the control SCC. To maintain the fresh concrete slump, HRWR was added. It was reported that due to the particle sizes of BA and RHA being larger than those of the OPC particles, the GCW absorbed more moisture. For these reasons, the mixtures of gSCCs needed more HRWR than the control SCC [28,29]. Moreover, the higher loss on ignition (LOI) of GCW and BA caused an increase in the requirements of the w/b ratio in the mixtures. In addition, the BA particles were characterized by a high porosity and were irregularly and angular shaped like RHA. For lubrication, gSCC prepared by BA needed a higher w/b ratio to sustain similar workability as compared to that of the control SCC. Additionally, the results agree with the research of Ali and Al-Tersawy [28].

##### Filling Ability

The filling ability was indicated by the slump time and flow to achieve a slump flow of 50.0 cm in diameter, which represented the SCC deformation under their weights against the surface frictions with no restraint. The time taken by SCC mixtures to achieve a 50.0 cm diameter (the slump flow time, T_50_) is linked to the flow rates. From Table 4, all the mixtures of the SCCs achieved an acceptable flow time that ranged from three to seven seconds. Using the GCW, the effect of the added GCW was to improve the gSCC viscosity due to incorporation of high-fineness particles generating a greater viscosity than the gSCC without GCW.

In mixtures with BA and RHA, the values of flow time were also measured. The addition of GCW in the mixtures helped in eliminating the mixing BA and RHA effects. With the higher GCW or lower OPC contents, the slump flow time decreased in the gSCC mixtures. This might be because of the smooth surfaces of the GCW that would increase the viscosity and inter-particle friction.

The V-funnel tests measured the time needed for SCCs to flow down via funnels. The V-funnel can be utilized to approximate the resistance to material segregations and the SCC paste viscosity. If the V-funnel time ranged between 8 and 12 s, the resistance to segregation was considered satisfactory for the SCC mixtures tested [13]. The research findings and results show clear correlations between the obtained differences in the levels of calcium carbonate substitution and V-funnel flow time results. As the proportion of fine aggregates and total binder contents substituted by RHA increased, the flow time increased. All the gSCC mixtures incorporating the GCW achieved V-funnel values within satisfactory ranges. The GCW effects can be accredited to particle fineness and the shape characteristics of the binder materials. Compared with coarse binders, fine binder materials improve the values of spread flow. By contributing to an increase in the free water amount, the smooth surface textures of the GCW improved these behaviors, which are known to also improve cohesiveness and workability.

##### Passing Ability

The gSCC mixture with BA and RHA agglomeration and higher viscosity could cause higher visible blocking. Utilizing BA and RHA to substitute for OPC had significant effects on the SCC passing ability as well as the filling ability. The GCW greatly affected the passing ability. A precise trend and pattern is shown by the values of the mean J-ring flow. As the GCW and OPC increased, the passing ability also improved. As the content of GCW increased, the mixtures showed noticeable blocking. This is because the total volume of the binder that should go through the confined voids decreased as the paste volumes of the gSCC mixture increased, increasing the friction of the inter-particles among the BA and RHA. Equally, the applications of GCW as ternary blends to generate gSCC mixtures improved the viscosity and helped minimize blocking [10].

### 3.2. Mechanical Properties

#### 3.2.1. Compressive Strength

**Figure 2:**
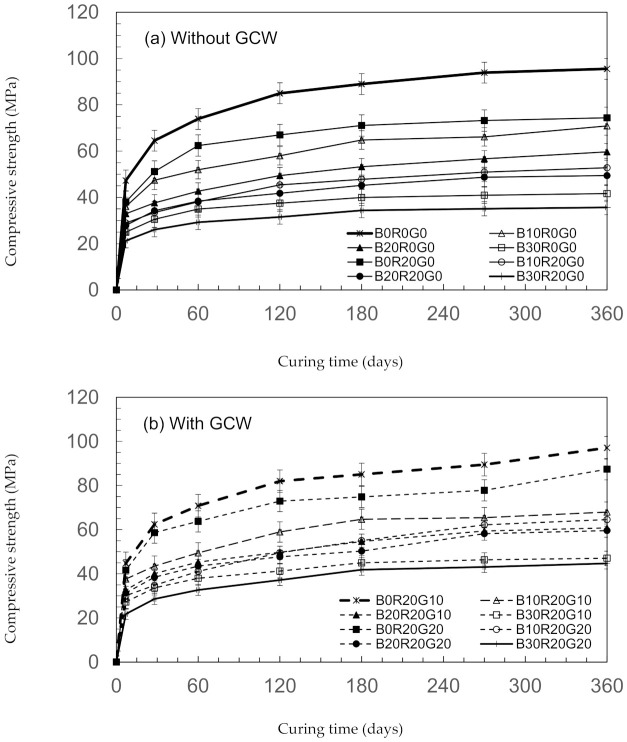
Compressive strengths of gSCC. (**a**) without GCW and (**b**) with GCW.

Compared to control gSCC (B0R0G0), the compressive strengths of the developments of the gSCC mixtures that mixed RHA with GCW are shown in Figure 2. At 28 days, with the maximum rate of compressive strength development, B0R20G10 had compressive strengths of 62.41 MPa. This later improved to 85.03 MPa and 97.13 MPa at 180 and 360 days, respectively. However, for the highest compressive strength for the mixture mixed with BA, the substitution with BA at 10 wt% (B10R20G10) also developed in strength but at a lower rate compared to without BA (B0R20G10). Compared to the control gSCC (B0R0G0), the findings show that the applications of the mixtures of GCW with BA and RHA as binder content decreased the concrete’s compressive strength at 360 days by about 28.85% (B10R20G10) and 32.41% (B10R20G20). This might be credited to the fact the compressive strengths of the concretes were derived partly from the pozzolanic reaction of RHA and BA and enhanced by the reaction of GCW [28]. These results agree with the findings of Aydın et al. [30] and Atis et al. [31]. The scholars noted that the compressive strengths of mortars using the binder content from the mixtures of RHA and GCW appeared to improve with age of curing in a way similar to the developments of the compressive strengths of OPC.

**Figure 3:**
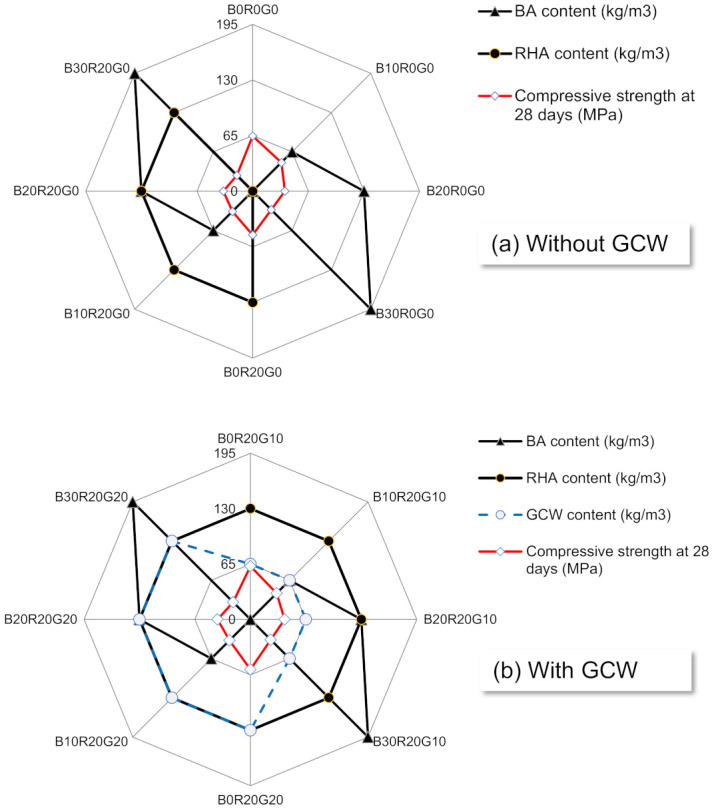
The relationships between compressive strengths of concretes and cement substitution levels of GCW, RHA, and BA in gSCC mixtures. (**a**) without GCW and (**b**) with GCW

The relationships between the compressive strengths and Portland cement substitution levels of BA/RHA/GCW in gSCC are indicated in Figure 3. The results show that utilizing 390 kg/m^3^ of OPC (B10R20G10) as a catalyst yielded compressive strengths of 10.04 MPa or 30.43% higher than those of B10R20G0 at 28 days. In general, a greater amount of OPC provides calcium hydroxide (Ca(OH)_2_) and C-S-H (calcium silicate hydrates), resulting in better pozzolanic and hydration reactions [32,33]. As compared to the control gSCC, B10R20G10 needed as much as 65 kg/m^3^ of GCW to achieve similar compressive strengths. The results and findings suggest that the applications of the mixtures of GCW and BA/RHA as binder materials might decrease the consumption of OPC by 40% [28]. Substituting 10% to 20% of the OPC was found to be optimal in comparison to the concretes integrating BA [12,34].

#### 3.2.2. Splitting Tensile Strength

**Figure 4:**
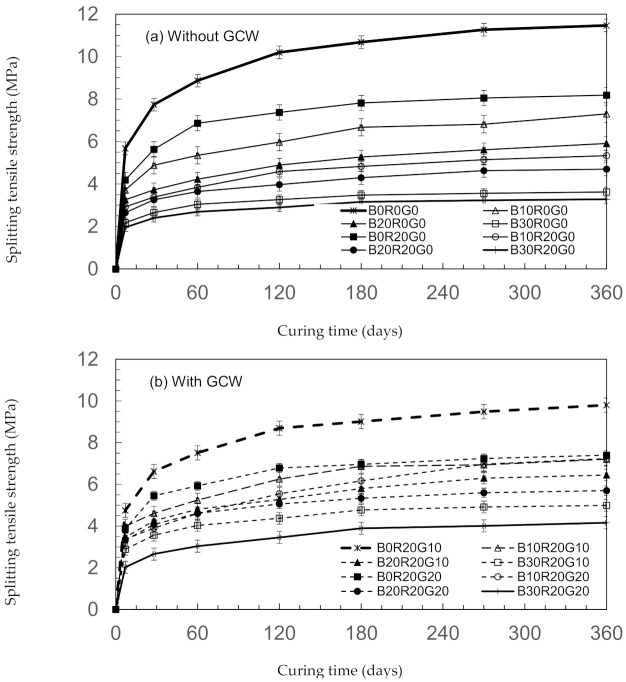
Splitting tensile strengths of gSCC. (**a**) without GCW and (**b**) with GCW.

With the improvement of compressive strengths, the splitting tensile strengths of B10R20G10 tended to improve compared to B10R20G0, as shown in Figure 4. These results show that the splitting tensile strengths are associated with the compressive strengths of the concretes [35]. The 28-day splitting tensile strength of B10R20G10, B20R20G10, and B30R20G10 were 43.5, 40.1, and 3.6 MPa, respectively. In addition, the splitting tensile strengths of gSCC tended to improve with all curing ages.

**Figure 5:**
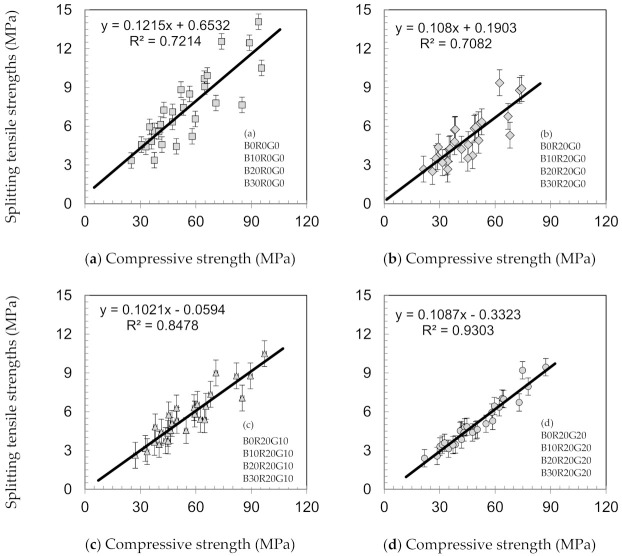
Relationships between splitting tensile strengths as a proportion of compressive strengths of concretes. (**a**) with BA, (**b**) with BA+20%RHA, (**c**) with BA+20%RHA+10%GCW, and (**d**) with BA+20%RHA+20%GCW.

As a proportion of compressive strengths, the splitting tensile strengths for gSCC are indicated in Figure 5. In gSCC, these percentages varied from 8.5 to 15%, which are the same as those of conventional concretes. Beixing et al. [27] noted that the proportions of the concrete splitting tensile strengths integrating BA were in the range of 10–12% of compressive strength. Additionally, the findings and results reinforce those of previous studies, showing that the plain concretes’ splitting tensile strengths were about ten percent of their compressive strengths. The results indicate that the splitting tensile strength increases linearly with increasing compressive strength [36].

#### 3.2.3. Moduli of Elasticity (MOE)

**Figure 6:**
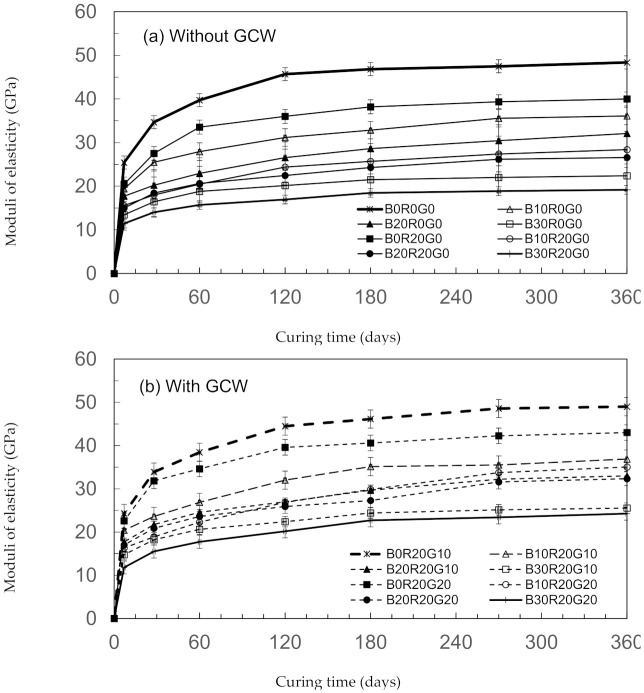
Moduli of elasticity of gSCC. (**a**) without GCW and (**b**) with GCW.

**Figure 7:**
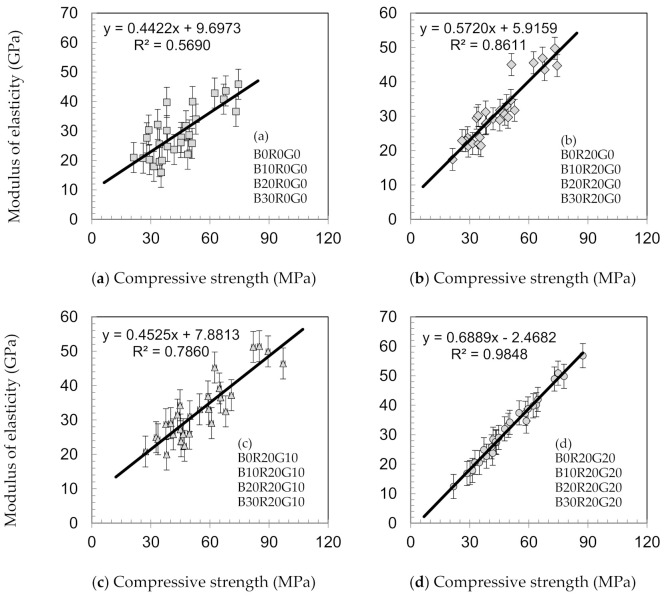
Relationships between the modulus of elasticity and compressive strength of gSCC. (**a**) with BA, (**b**) with BA+20%RHA, (**c**) with BA+20%RHA+10%GCW, and (**d**) with BA+20%RHA+20%GCW.

The elasticity moduli of the concretes over 360 days of testing are shown in Figure 6. At 28 days, the elasticity moduli of B0R20G0, B10R20G0, B20R20G0, and B30R20G0 are 27.5, 18.2, 18.1, and 14.2 GPa, respectively. The elasticity moduli at 360 days are 36.9, 32.3, and 25.5 GPa for B10R20G10, B20R20G10, and B30R20G10, respectively, as shown. The findings show that the elasticity moduli of gSCC with GCW appear to improve with curing ages. Figure 7 shows the relationships between the modulus of elasticity and compressive strength of gSCC. The elasticity moduli were virtually associated with the strengths of the concretes. The results and findings are in accordance with those of the studies of Chen et al. [37] and Chindaprasirt et al. [38]. The scholars noted that the elasticity moduli of concretes having pozzolanic substances, such as virgin pozzolans and fly ashes, appeared to improve with curing age in a similar manner to the moduli of elasticity developments of traditional concretes [38].

## 4. Optimal Content of GCW

**Figure 8:**
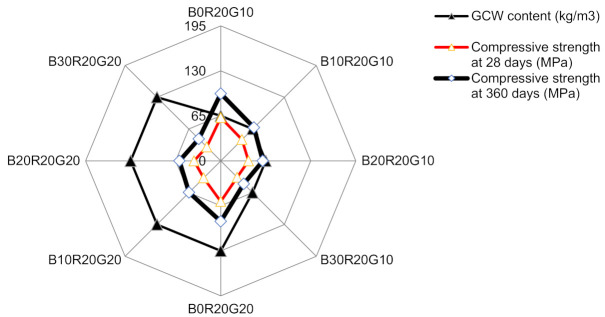
Relationships between compressive strengths of concretes and cement substitution levels of GCW in gSCC mixtures.

The gSCC specimens were utilized to establish the optimal percentages of GCW associated with BA and RHA [3]. At a constant of 1:2.15, a binder to aggregate mortars was set by mass as outlined by ASTM C39 [24]. By adjusting the content of water in the gSCC, the flow of gSCC was sustained with the controlled slump flow of a 70.0 ± 2.5 cm diameter [13]. The gSCC binder contents were mixtures of BA, RHA, and GCW. The OPC was substituted by BA, RHA, and GCW at percentage replacements of 40 wt% (B10R20G10) to 70 wt% (B30R20G20) by mass of the total binder content of 650 kg/m^3^. Until the testing stage, the gSCCs were cured in saturated lime water (calcium hydroxide) [39,40,41]. For each stage, the mean of compressive strengths of mortars was obtained from three samples. The relationships between compressive strengths and the substitution of GCW with OPC in the BA and RHA mixtures at the rates of 10 and 20% by mass of the total binder contents incorporating BA, RHA, and GCW are presented in Figure 8. It was demonstrated that the 28-day and 360-day compressive strengths of B10R20G10 improved from 10.1 to 33.5 MPa and from 10.2 to 52.8 MPa. The optimum ratio of BA plus RHA to GCWs was 67:33 by mass. This ratio yielded the greatest compressive strength of 37.6 MPa at 7 days and 43.5 MPa at 28 days. 

Compared to gSCC that required 390 kg/m^3^ of OPC, the gSCC prepared by 10 wt% GCW associated with 10 wt% BA and 20 wt% RHA was improved significantly. The filling and passing abilities of the gSCC were improved by using GCW. In addition, the gSCC achieved continuous mechanical development compared to the control SCC, as the binder contents could minimize the consumption of OPC by up to 40%. 

At present, there is no economically feasible mineral that can produce manufactured cement with an equivalent quality to that of the present OPC [14]. Researchers have studied the impact of the substitutions by adding minerals to the mechanical features of cements and decreasing the emission of CO_2_. The investigations state that by decreasing OPC by replacing it with different pozzolanic materials and using raw materials for OPC production, CO_2_ emissions may easily be decreased [16]. The researchers investigated the energy that contributes to the use of technology to reduce CO_2_ emissions. They used life-cycle assessing techniques to distinguish the freshly installed and available technology and also the ready-mixed concrete plant. The researchers suggest the potential of computational fluid dynamics (CFDs) to help optimize the suitable mix proportions for gSCC based on the fresh state behaviors and to help reduce CO_2_ emissions from the production of concrete.

## 5. Statistically Descriptive and ANOVA Analyses of the gSCC Mixtures Based on Compressive Strength Development (CSD)

Table 5 shows the normalized CSD of gSCC mixtures compared to the control SCC. Table 6 shows the results of the single-factor ANOVA analysis of normalized CSD compared to the control SCC (B0R0G0), obtained from Table 5, with the assumptions including a normally distributed population, an equal variance population, and an independently sampled population. Part (a) indicates the influence of BA on gSCC without RHA and GCW, *p*-value (1.86627 × 10^−10^) < 0.01 at α= 0.05, which means that the incorporation of an increasing percentage of replacement BA, from 10 to 30 wt%, in OPC yielded a statistically significant difference, indicating a reasonable effect on the CSD of the gSCC. These results can be interpreted similarly to those of the CSD of (b) gSCC containing 20 wt% of RHA and without GCW (*p*-value (3.79911 × 10^−8^) < 0.01). In addition to the significance of the interpretation of the ANOVA results, the lower replacement amount of BA in OPC yielded a higher CSD, and the lowest CSD of gSCC occurred for the B30R0G0 (without RHA) and B30R20G0 (with RHA) mixtures.

The influence of the GCW on the properties of the gSCC prepared by BA and RHA are presented in Table 6. Parts (c), (d), and (e), show the influence of GSW in gSCC mixed with 10, 20, and 30 wt% of BA and 20 wt% of RHA, respectively. It was demonstrated that incorporating the GCW in the gSCC mixtures had a significant effect on the CSD up to 360 curing days. For example, the gSCC mixed with 10% BA and 20 wt% RHA had a *p*-value (4.90469 × 10^−6^) < 0.01, resulting in a statistically significant difference, indicating a reasonable effect on the CSD of the gSCC as well as in the mixtures of the gSCC mixed with 20% BA and 20 wt% RHA (*p*-value of 4.66498 × 10^−5^) and 30% BA and 20 wt% RHA (*p*-value of 2.424 × 10^−7^).

Referring to the descriptive meaning of the CSD of the gSCC in Table 6, parts (c), (d), and (e), it was found that the average values of the gSCC mixtures containing 10 wt% GCW (B10R20G10, B20R20G10 and B30R20G10) had the highest CSD compared to those mixtures mixed with 0 and 20 wt% GCW. This is due to the filling effect of GCW particles, consistent with a continuous increase in CSD over 360 days. However, the variances of the gSCC mixtures containing 10 wt% GCW were also higher than those of 0 and 20 wt% GCW, possibly due to the physical variance effects between the filling effect and the amount of OPC hydration (C-S-H) products, while the 20 wt% GCW was affected by a decrease in OPC content, resulting in a lower amount of hydration product.

Compared to the gSCC mixtures containing GCW, the B10R20G10 mixture with 10 wt% BA, 20 wt% RHA, and 10 wt% GCW in replacement of 40 wt% of OPC yielded the greatest CSD over 360 days of curing due to the particle filling effect associated with the hydrated level of OPC and the hydration products as mentioned above.

## 6. Conclusions

Based on the experimental and analysis results from the investigation of using GCW in gSCC production prepared by triple combined GCW (10 and 20 wt%), low-quality BA (10, 20, and 30 wt%) and low-quality RHA (20 wt%), the following conclusions can be made:

- In order to maintain the controlled slump flows of gSCC at a 70.0 ± 2.5 cm diameter, the w/b ratios of the gSCC mixtures incorporating BA and RHA varied in the range between 0.48 and 0.54 and 0.49 and 0.56, respectively. The required w/b ratios for the gSCC with BA and RHA were greater than those for SCCs without RHA. With BA and RHA incorporating GCW, the gSCC had lower w/b ratios than those without BA and RHA, but had higher w/b ratios than the control SCCs.
- When the amounts of GCW increased, the fresh densities of the gSCC mixtures incorporating BA and RHA decreased with a GCW content of 10 wt%. The fresh densities of the gSCC with mixtures of RHA also decreased when increasing the amount of GCW.
- The majority of the mixtures retained acceptable flow times considering the SCC workability. The differences in the levels of GCW substitution and the V-funnel flow results showed general correlations with the increase in GCW. In addition, gSCC prepared by BA needed more w/b to sustain similar workability as compared to that of the control SCC.
- The mechanical performance of the gSCC continuously developed in comparison to the control SCC, as the binder contents could minimize the consumption of OPC by up to 40%. The gSCC mixtures of the GCW with BA and RHA as binder decreased the concrete’s compressive strength at 360 days.
- The greatest compressive strength was achieved in the mixtures of the gSCC prepared with 10 wt% GCW in 10 wt% BA and 20 wt% RHA, which were consistent with descriptive and ANOVA analyses of the gSCC mixtures based on compressive strength development.
- Compared to the gSCC that required 390 kg/m^3^ of OPC, the gSCC prepared with 10 wt% GCW associated with 10 wt% BA and 20 wt% RHA was improved, with the properties of workability including filling and passing abilities.

## Figures and Tables

**Table 1:** Physical properties and pozzolanic activity of the materials.

Materials	Mean Particle Size (µm) (Average ± SD)	Specific Gravity (Average ± SD)	Retained on a 45 µm Sieve (No.325) (%) (Average ± SD)	Pozzolanic Actvity (mg CaO/g Sample) (Average ± SD)
OPC	14.61 ± 1.38	3.14 ± 0.02	3.53 ± 0.98	None
BA	25.70 ± 4.22	2.27 ± 0.01	14.53 ± 1.02	179.44 ± 8.24
RHA	17.31 ± 2.56	2.32 ± 0.01	10.06 ± 3.11	433.29 ± 1.39
GCW	3.02 ± 0.26	2.42 ± 0.01	1.12 ± 0.34	512.85 ± 10.26

**Table 2:** Chemical components of OPC, BA, RHA, and GCW.

Chemical Components (% by mass)	OPC (Mean ± SD)	BA (Mean ± SD)	RHA (Mean ± SD)	GCW (Mean ± SD)
Loss on ignition (LOI)	0.92 ± 0.05	19.62 ± 3.06	6.31 ± 0.72	36.16 ± 2.11
Sulfur tri-oxide (SiO_3_)	2.72 ± 0.02	2.27 ± 0.23	0.03 ± 0.01	0.12 ± 0.02
Potassium oxide (K_2_O)	0.31 ± 0.01	1.21 ± 0.19	1.34 ± 0.03	0.11 ± 0.01
Sodium oxide (Na_2_O)	0.22 ± 0.01	0.25 ± 0.02	0.12 ± 0.01	0.21 ± 0.01
Magnesium oxide (MgO)	1.26 ± 0.02	0.93 ± 0.04	0.30 ± 0.02	1.72 ± 0.03
Calcium oxide (CaO)	65.83 ± 3.82	11.49 ± 1.87	0.92 ± 0.04	56.53 ± 8.19
32Iron oxide (Fe_2_O_3_)	3.44 ± 0.12	4.12 ± 0.32	0.14 ± 0.01	0.98 ± 0.07
Aluminum oxide (Al_2_O_3_)	4.78 ± 0.06	5.16 ± 0.44	0.47 ± 0.02	0.42 ± 0.06
Silicon dioxide (SiO_2_)	21.99 ± 2.35	55.06 ± 5.23	91.42 ± 6.81	4.38 ± 0.18

**Table 3:** Mix proportions of gSCC.

Mixture	Binder (kg/m^3^)	OPC (kg/m^3^)	BA (kg/m^3^)	RHA (kg/m^3^)	GCW (kg/m^3^)	Sand (kg/m^3^)	Crushed Limestone (kg/m^3^)	w/b	HRWR (%)
B0R0G0 ^[1]^ (Control SCC)	650	650	0	0	0	737	657	0.40	2.04
B10R0G0	650	585	65	0	0	737	657	0.48	2.04
B20R0G0	650	520	130	0	0	737	657	0.50	2.04
B30R0G0	650	455	195	0	0	737	657	0.54	2.04
B0R20G0	650	520	0	130	0	737	657	0.45	2.04
B10R20G0	650	455	65	130	0	737	657	0.49	2.04
B20R20G0	650	390	130	130	0	737	657	0.51	2.04
B30R20G0	650	325	195	130	0	737	657	0.56	2.04
B0R20G10	650	455	0	130	65	737	657	0.42	2.04
B10R20G10	650	390	65	130	65	737	657	0.45	2.04
B20R20G10	650	325	130	130	65	737	657	0.48	2.04
B30R20G10	650	260	195	130	65	737	657	0.49	2.04
B0R20G20	650	390	0	130	130	737	657	0.43	2.04
B10R20G20	650	325	65	130	130	737	657	0.44	2.04
B20R20G20	650	260	130	130	130	737	657	0.45	2.04
B30R20G20	650	195	195	130	130	737	657	0.47	2.04

Remarks: ^[1]^ BXRYCZ denotes the following: BX is the percentage OPC replacement of the BA content (0, 10, 20, and.30 wt%), RY is the percentage OPC replacement of the RHA content (0 and 20 wt%), and GZ is the percentage OPC replacement of GCW (0, 10, and 20 wt%).

**Table 4:** Workability of the gSCC mixtures.

Mixture	Targeted Flow Slump (cm)	T_50_ (s)	J−Ring Test (Initial) (cm)	Difference (Initial) (cm)	Blocking Assessment ^[1]^	V-Funnel Flow Time (s)	Fresh Density	
							Value (kg/m^3^)	% Control
B0R0G0	67.5	2	67.0	0.5	Not visible	6	2483.2	100.00
B10R0G0	65.5	3	63.5	2.0	Not visible	8	2454.6	−1.15
B20R0G0	65.0	4	62.5	2.5	Noticeable	18	2425.9	−2.31
B30R0G0	63.5	5	58.0	5.5	Extreme blocking	45	2397.3	−3.46
B0R20G0	66.5	4	64.5	2.0	Not visible	10	2427.2	−2.26
B10R20G0	64.5	5	61.0	3.5	Noticeable	14	2398.5	−3.41
B20R20G0	63.5	6	57.0	6.5	Extreme blocking	36	2369.9	−4.56
B30R20G0	62.5	7	55.0	7.5	Extreme blocking	82	2341.3	−5.72
B0R20G10	67.5	3	66.5	1.0	Not visible	8	2459.4	−0.96
B10R20G10	67.0	5	65.5	1.5	Not visible	10	2430.8	−2.11
B20R20G10	66.5	5	64.5	2.0	Not visible	11	2402.1	−3.26
B30R20G10	66.0	6	62.5	3.5	Noticeable	12	2373.5	−4.42
B0R20G20	66.5	2	64.5	2.0	Not visible	4	2432.5	−2.04
B10R20G20	65.0	3	62.5	2.5	Noticeable	6	2403.9	−3.19
B20R20G20	65.0	3	60.5	4.5	Noticeable	9	2375.3	−4.35
B30R20G20	64.0	4	59.0	5.0	Noticeable	12	2346.6	−5.50

Remark: ^[1]^ Complying with ASTM C1621 [21].

**Table 5:** Normalized CSD compared to the control SCC (B0R0G0).

(**a**) gSCCs prepared without GCW									
	**Mixture**	**B0R0G0**	**B10R0G0**	**B20R0G0**	**B30R0G0**	**B0R20G0**	**B10R20G0**	**B20R20G0**	**B30R20G0**
**Age**									
0 day		0.00000	0.00000	0.00000	0.00000	0.00000	0.00000	0.00000	0.00000
7 days		1.00000	0.76276	0.69624	0.52832	0.80533	0.61193	0.59019	0.44963
28 days		1.00000	0.73490	0.58304	0.47351	0.79332	0.51898	0.53125	0.40411
60 days		1.00000	0.70308	0.57759	0.47335	0.84439	0.51529	0.51959	0.39547
120 days		1.00000	0.68225	0.58128	0.44109	0.78817	0.53390	0.49187	0.37090
180 days		1.00000	0.72832	0.59831	0.44943	0.79861	0.53736	0.50801	0.38617
270 days		1.00000	0.70458	0.60326	0.43612	0.77934	0.54223	0.51844	0.37397
360 days		1.00000	0.74173	0.62444	0.43587	0.77852	0.55265	0.51743	0.37324
(**b**) gSCCs prepared with GCW									
	**Mixture**	**B0R20G10**	**B10R20G10**	**B20R20G10**	**B30R20G10**	**B0R20G20**	**B10R20G20**	**B20R20G20**	**B30R20G20**
**Age**									
0 day		0.00000	0.00000	0.00000	0.00000	0.00000	0.00000	0.00000	0.00000
7 days		1.00000	0.94688	0.79551	0.69052	0.57553	0.88060	0.63641	0.66290
28 days		1.00000	0.96784	0.67467	0.62156	0.52130	0.90977	0.53974	0.59670
60 days		1.00000	0.95880	0.66987	0.61312	0.51411	0.86292	0.55599	0.58859
120 days		1.00000	0.96493	0.69407	0.58533	0.48588	0.85879	0.58302	0.56192
180 days		1.00000	0.95587	0.72780	0.61469	0.50588	0.84117	0.61863	0.56551
270 days		1.00000	0.95312	0.69705	0.63250	0.49364	0.82922	0.66220	0.61985
360 days		1.00000	1.01657	0.71153	0.63643	0.49267	0.91492	0.67596	0.62371

**Table 6:** Descriptive and one-way ANOVA analysis results at α = 0.05 obtained from normalized compressive strength compared to the control SCC (B0R0G0).

(**a**) Influence of BA in gSCC (without RHA and GCW)						
**Mixture**	**Count**		**Sum (−)**	**Average (−)**		**Variance (−)**
B10R0G0	7		5.057620997	0.722517285		0.000749467
B20R0G0	7		4.264155899	0.609165128		0.001737440
B30R0G0	7		3.237675198	0.462525028		0.001100864
Source of Variation	SS	df	MS	F	*p*-value	F crit
Between Groups	0.237878676	2	0.118939338	99.4539509	1.86627 ×10^−10^	3.554557146
Within Groups	0.021526627	18	0.001195924	-	-	-
(**b**) Influence of BA in gSCC mixed with 20 wt% RHA (without GCW)						
**Mixture**	**Count**		**Sum (−)**	**Average (−)**		**Variance (−)**
B10R20G0	7		3.812343666	0.544620524		0.001047734
B20R20G0	7		3.676774698	0.525253528		0.000967302
B30R20G0	7		2.753489456	0.393355637		0.000770578
Source of Variation	SS	df	MS	F	*p*-value	F crit
Between Groups	0.094857468	2	0.047428734	51.07894687	3.79911 × 10^−8^	3.554557146
Within Groups	103643.7358	24	4318.488992	-	-	-
(**c**) Influence of GSW in gSCC mixed with 10 wt% BA and 20 wt% RHA						
**Mixture**	**Count**		**Sum (−)**	**Average (−)**		**Variance (−)**
B10R20G0	7		3.812343666	0.544620524		0.001047734
B10R20G10	7		4.970509880	0.710072840		0.001818456
B10R20G20	7		4.271944572	0.610277796		0.002737935
Source of Variation	SS	df	MS	F	*p*-value	F crit
Between Groups	0.09717026	2	0.04858513	26.00858778	4.90469 × 10^−6^	3.554557146
Within Groups	0.033624753	18	0.001868042	-	-	-
(**d**) Influence of GSW in gSCC mixed with 20 wt% BA and 20 wt% RHA						
**Mixture**	**Count**		**Sum (−)**	**Average (−)**		**Variance (−)**
B20R20G0	7		3.676774698	0.525253528		0.000967302
B20R20G10	7		4.394147722	0.627735389		0.001043431
B20R20G20	7		4.219173057	0.602739008		0.001273434
Source of Variation	SS	df	MS	F	*p*-value	F crit
Between Groups	0.039973151	2	0.019986575	18.25720982	4.66498 × 10^−5^	3.554557146
Within Groups	0.019705002	18	0.001094722	-	-	-
(**e**) Influence of GSW in gSCC mixed with 30 wt% BA and 20 wt% RHA						
**Mixture**	**Count**		**Sum (−)**	**Average (−)**		**Variance (−)**
B30R20G0	7		2.753489456	0.393355637		0.000770578
B30R20G10	7		3.589017400	0.512716771		0.000925573
B30R20G20	7		3.180552613	0.454364659		0.000178851
Source of Variation	SS	df	MS	F	*p*-value	F crit
Between Groups	0.049873018	2	0.024936509	39.89837688	2.424 × 10^−7^	3.554557146
Within Groups	0.01125001	18	0.000625001	-	-	-

Remark: The compressive strengths of control SCC were 47.30, 64.50, 73.90, 84.97, 89.00, 93.90, and 95.54 MPa at 7, 28, 60, 120, 180, 270, and 360 days (Count = 7), respectively.

## Data Availability

The data presented in this study are available on request from the corresponding author.
